# Implant therapy for a patient with osteogenesis imperfecta type I: review of literature with a case report

**DOI:** 10.1186/s40729-018-0148-0

**Published:** 2018-11-23

**Authors:** Shamit S. Prabhu, Kevin Fortier, Michael C. May, Uday N. Reebye

**Affiliations:** 1 Wake Forest School of Medicine, Winston-Salem, USA; 2 Boston University Henry M. Goldman School of Dental Medicine, Boston, USA; 3 Virginia Commonwealth University School of Dentistry, Richmond, USA; 4 Triangle Implant Center, 5318 NC Highway 55, Suite 106, Durham, NC 27713 USA

**Keywords:** Osteogenesis imperfecta type I, Implant therapy, Brittle bone disease

## Abstract

Bone fragility and skeletal irregularities are the characteristic features of osteogenesis imperfecta (OI). Many patients with OI have weakened maxillary and mandibular bone, leading to poor oral hygiene and subsequent loss of teeth. Improvements in implant therapy have allowed for OI patients to achieve dental restoration. However, there is limited available literature on implant therapy for patients with OI. The greatest challenge in the restoration process for OI patients in an outpatient setting is ensuring primary stability and osseointegration. Improvements in synthetic grafts improve successful implant placement and prevent predisposing patients to unnecessary procedures. This report details the successful restoration process of an OI type I patient’s maxillary arch in addition to a review of the currently available literature.

## Introduction

Osteogenesis imperfecta (OI), colloquially known as “brittle bone disease,” is a broad term for a group of congenital disorders affecting the connective tissue resulting in a susceptibility to fractures. In 1979, Sillence et al. conducted an epidemiological and genetic study of OI patients [1]. These patients were grouped according to four distinct syndromes: (1) dominantly inherited OI with blue sclerae, (2) lethal perinatal OI with radiographically crumpled femora and beaded ribs, (3) progressively deforming OI with normal sclerae, or (4) dominantly inherited OI with normal sclerae [1]. These groupings would later become the clinical features in identifying OI types I–IV. Since then, additional types of OI have been classified based on allelic heterogeneity, histological variance, radiological features, and clinical manifestations (Table 1).

**Table 1:** Osteogenesis imperfecta classifications

Type	Inheritance	Gene	Locus	Clinical features	OMIM
I [1, 4]	AD	*COL1A1* or *COL1A2*	17q21.33 or 7q21.3	Variable bone fragility, moderate bone deformity, blue sclerae, possible dentinogenesis imperfecta	166,200
II [1, 26]	AD	*COL1A1* or *COL1A2*	17q21.33 or 7q21.3	Perinatally lethal	166,210
III [1, 27]	AD	*COL1A1* or *COL1A2*	17q21.33 or 7q21.3	Severe bone fragility, progressively deforming, normal sclerae, dentinogenesis imperfecta, cardiovascular complications, spinal curvature, kyphoscoliosis	259,420
IV [1]	AD	*COL1A1* or *COL1A2*	17q21.33 or 7q21.3	Moderate bone fragility, moderate deformity, normal sclerae, short stature, possible dentinogenesis imperfecta, kyphoscoliosis	166,220
V [28, 29]	AD	*IFITM5*	11p15.5	Moderate to severe bone fragility, radial head dislocation, normal to blue sclerae, normal dentin	610,967
VI [30]	AR	*SERPINF1*	17p13.3	Moderately to severe deformity, fish-scale pattern of lamellae, excessive osteoid, normal dentin	613,982
VII [31]	AR	*CRTAP*	3p22.3	Severe bone fragility, progressively deforming, normal sclerae, severe rhizomelia and coxa vera, normal dentin	610,682
VIII [32]	AR	*LEPRE1*	1p34.2	Severe bone fragility, normal sclerae, bulbous metaphyses, round face, short barrel-shaped chest	610,915
IX [33]	AR	*PPIB*	15q22.31	Severe bone deformity, gray sclerae	259,440
X [34]	AR	*SERPINH1*	11q13.5	Multiple bone deformities and fractures, osteopenia, dentinogenesis imperfecta, blue sclerae	613,848
XI [35]	AR	*FKBP10*	17q21.2	Mild to severe bone deformity, normal to gray sclerae	610,968
XII [36]	AR	*SP7*	12q13.13	Mild bone deformity, normal dentin, normal hearing, normal sclerae	613,849
XIII [37]	AR	*BMP1*	8p21.3	Severe growth deficiency, severe bone deformity, normal dentin, light blue sclerae	614,856
XIV [38]	AR	*TMEM38B*	9q31.2	Variable bone deformity, variable osteopenia, normal dentin, normal sclerae, normal hearing	615,066
XV [39, 40]	AR	*WNT1*	12q13.12	Severe bone deformity, short stature, early and recurrent fractures, normal dentin, possible blue sclerae, normal hearing	615,220
XVI [41]	AR	*CREB3L1*	11p11.2	Severe bone deformity, beaded ribs, callus formation, cardiac irregularities	616,229
XVII [42]	AR	*SPARC*	5q33.1	Progressive severe bone fragility, kyphoscoliosis, mild joint hyperlaxity, short stature	616,507

*AD* autosomal dominant, *AR* autosomal recessive, *OMIM* Online Mendelian Inheritance in Man

In studies conducted in Europe and the US, the birth prevalence of OI was estimated to be 0.3–0.7 per 10,000 births [2, 3]. Incidence in males and females is roughly equal. The pathophysiology for OI type I is characterized by mutations in the genes for proα1 chains on *COL1A1* on chromosome 17 or for proα2 chains on *COL1A2* on chromosome 7 [4]. The prominence of type I collagen in the extracellular matrix of bones and skin results in patients with OI having qualitative or quantitative defects. In OI type I, individuals have quantitative defects in their normal type I collagen in that the collagen is functionally normal but produced in smaller quantities. Individuals with qualitative defects produce structurally defective type I collagen resulting in moderate deformations as seen in OI type IV, to severe deformation as seen in OI type III, and can even be lethal in OI type II [5].

Clinically, patients with OI type I present with an increased risk of bone fractures due to fragile bone, osteoporosis, blue sclerae, short stature, joint hypermobility, and susceptibility to conductive hearing loss progressing from adolescence to adulthood [6]. OI type I can be further categorized based on the presence, Ia, or absence, Ib, of dentinogenesis imperfecta (DI) [7]. Patients with DI will have opalescent teeth due to abnormal dentin exposure through the translucent enamel with a variable blue-gray or yellow-brown hue. Radiographical features of dentinogenesis imperfecta include deposition of dentin resulting in a marked reduction of the pulp chamber and root canals, short roots with constricted corono-radicular junctions, and bulbous crowns [8, 9]. Improper dentin formation predisposes patients to an increased risk of dental fractures and increased wear on teeth, subsequently requiring corrective dental procedures [10]. Navigating treatment options for patients with OI type I pose many challenges for dental professionals. In particular, successful dental implant treatment is difficult to achieve due to requiring strong, dense bone for acceptance of the implant. To avoid implant failure, patients must maintain routine oral care in addition to closely monitoring bone healing around the implant site. Implant treatment is even more challenging if the patient is prescribed bisphosphonates. These drugs are often administered to reduce osteoclast activity to limit bone resorption, subsequently improve bone microarchitecture, and bone density and correct vertebral size and shape [11–13]. One randomized, double-blind, placebo-controlled trial on the effectiveness of Risedronate in children with OI showed a significant reduction in the risk of fractures [13]. While bisphosphonate treatment may assist in the prevention of long bone fractures, it can be a detriment in the oral restoration process.

In this report, we focus on the 3-year dental implant therapy and restorative process of a 53-year-old male patient diagnosed with OI type I.

## Case presentation

### Evaluation

**Fig. 1:**
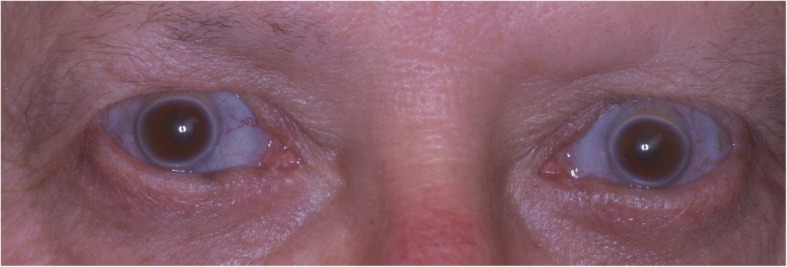
Characteristic blue sclerae

A 53-year-old male diagnosed with OI type I was referred to our clinic for extraction of the remaining maxillary teeth and evaluation for full arch immediate load hybrid prosthesis. His clinical history included osteogenesis type 1, bipolar disorder, alopecia, and hypothyroidism. The patient presented with normal stature, measuring 170.18 cm and weighing 81.65 kg with characteristic blue sclerae of OI type I (Fig. 1). Throughout his life, he has had multiple orthopedic fractures due to his OI. At the time of surgery, he was on Lamictal, Xarelto, Synthroid, lisinopril, and hydrochlorothiazide.

**Fig. 2:**
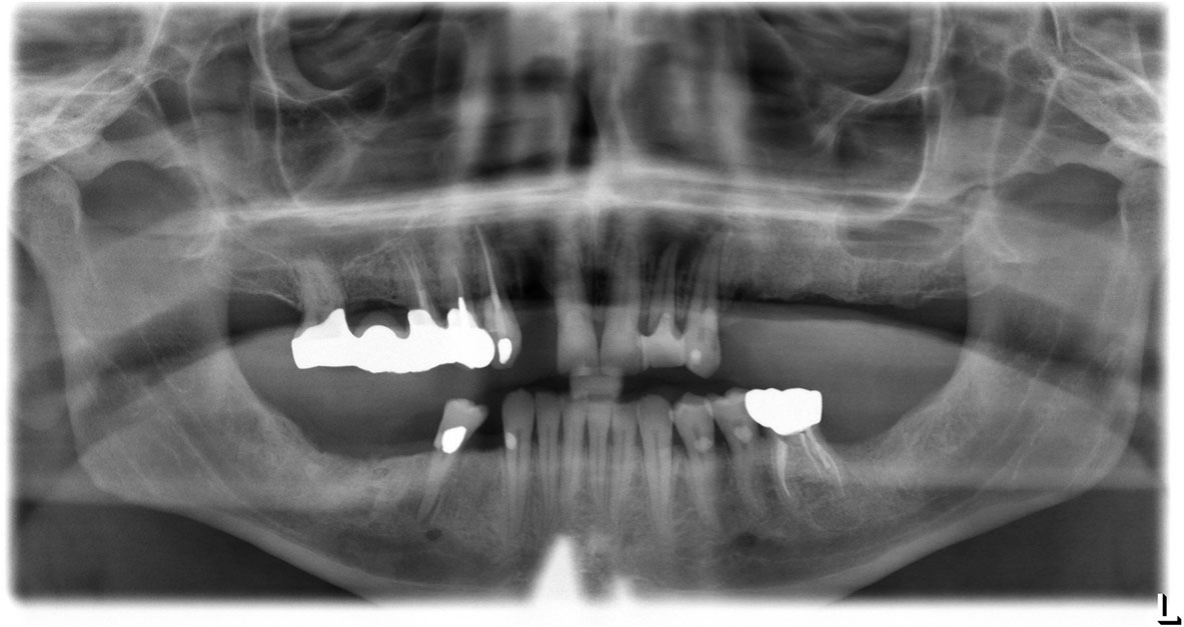
Pre-operative panoramic radiograph

Extraoral, TMJ, intraoral soft tissue, and lymph node examinations produced no abnormal findings. An examination of the dentition revealed the maxillary teeth were in poor repair with a fixed bridge extending from site number 2 to site number 5 with site number 3 serving as the pontic abutment. Sites number 8, number 9, number 10, and number 11 have periodontal involvement as well as recurrent decay. He was edentulous on the posterior left maxillary arch. His lower dentition consisted of sites number 19 through number 27 with number 28 being edentulous and number 29 having a root fracture (Fig. 2). The upper jaw had good ridge width with reproducible centric relation and centric occlusion. The patient was otherwise healthy apart from medical issues directly related to his OI.

Due to his significant gag reflex, he was unable to wear a removable prosthesis. Lengthy conversations regarding implant therapy and implant options were reviewed as well as risks with his OI. Options presented included no treatment, placement of fixtures to support a removable prosthesis, placement of fixtures to support a fixed hybrid, and placement of axial implants for fixed denture prosthesis. He elected for a fixed denture prosthesis. Our patient was apprehensive towards having full edentulation and implant placement completed all at once and decided to have the implants placed in stages (Table 2).

**Table 2:** Chronological timeline of the implant therapy of the maxilla

Date	Site number	Implant diameter (mm)	Implant length (mm)	Immediate load	Bone graft augmentation
3/26/14	12	4.3	10	Yes	Allograft
3/26/14	14	4.3	10	Yes	None
11/10/14	10	3.5	13	Yes	Allograft
3/5/15	7	3.5	13	Yes	Allograft
4/19/16	11	4.3	11.5	Yes	None
2/22/17	3	4.3	10	Yes	Allograft
2/22/17	4	5.0	10	Yes	Allograft
2/22/17	6	4.3	13	Yes	Allograft
2/22/17	8	3.5	10	Yes	Allograft
2/22/17	9	3.5	10	Yes	Allograft

### Surgical technique

The patient underwent implant therapy in stages under general anesthesia with immediate load protocol. Intravenous access was obtained, and the patient was anesthetized under general anesthesia by our anesthesiologist. Carpules of 2% lidocaine with 1:100,000 epinephrine, 4% articaine hydrochloride with 1:100,000 epinephrine (Septocaine), and 0.5% bupivacaine hydrochloride with 1:200,000 epinephrine (Marcaine) were used as needed. For each site, a 15 blade was used to make a sulcular incision from the mesial to the distal aspect of the tooth. A full thickness mucoperiosteal flap was elevated with a periosteal elevator exposing the buccal alveolus. Buccal bone was removed using a surgical fissure bur to allow for osteotomes and elevators to atraumatically elevate and deliver the teeth, while preserving lingual, mesial, and distal walls. Next, a straight elevator was positioned between the alveolus and the root surface. The tooth was elevated, and the periodontal ligament was separated from the alveolus. The tooth was extracted using a no. 150 upper universal forcep. The socket was curetted and irrigated with copious amounts of normal saline solution. A bone file and rongeur were used to smoothen the alveolus.

To deliver implants, all bony walls were checked with a perio probe to verify the depth. A series of osteotomy burs were used at 1000 RPM and 50 Ncm of torque with copious sterile normal saline irrigation. At each step, angulation was checked. Once the final osteotomy was completed, the site was checked to verify that all bony walls were stable. A NobelActive implant was torqued into position at greater than 30 Ncm followed by placement of a cover screw. In instances where grafting was necessary, the graft material was positioned to obliterate the bony defect using a periosteal elevator and curette to place in the bony voids. The gingival tissues were repositioned using an Adson Tissue Forcep. A tension-free closure was attained with a periosteal release technique. The sites were closed with interrupted 3-0 gut sutures. All procedures were accomplished without any further complications.

### Prosthetic procedure

**Fig. 3:**
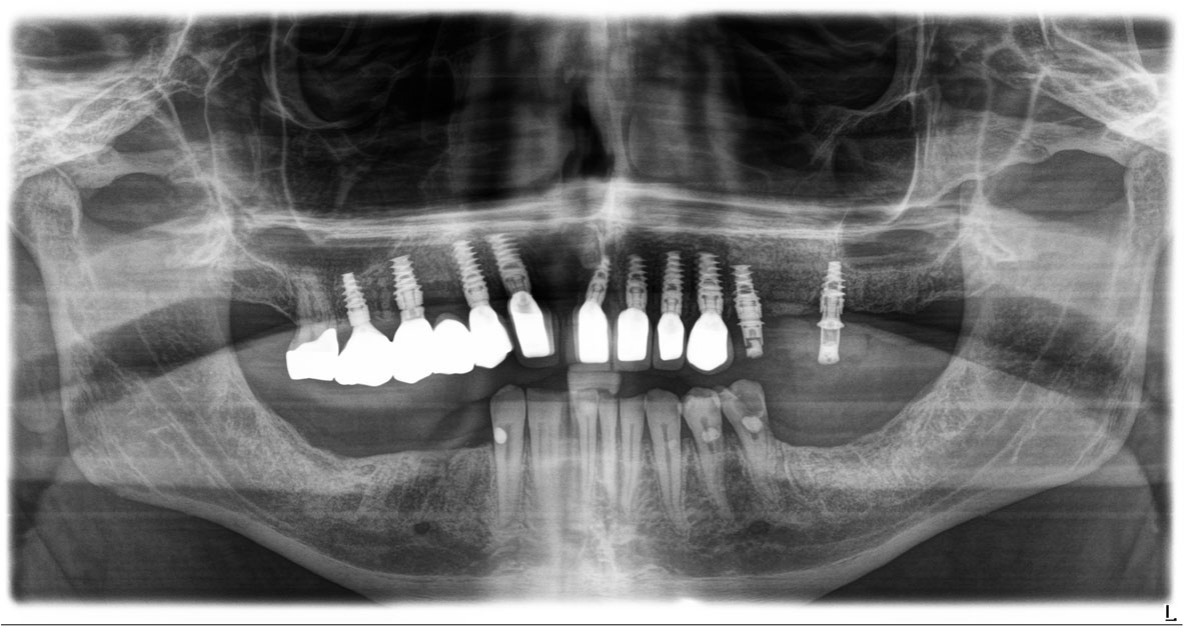
Post-operative panoramic radiograph

**Fig. 4:**
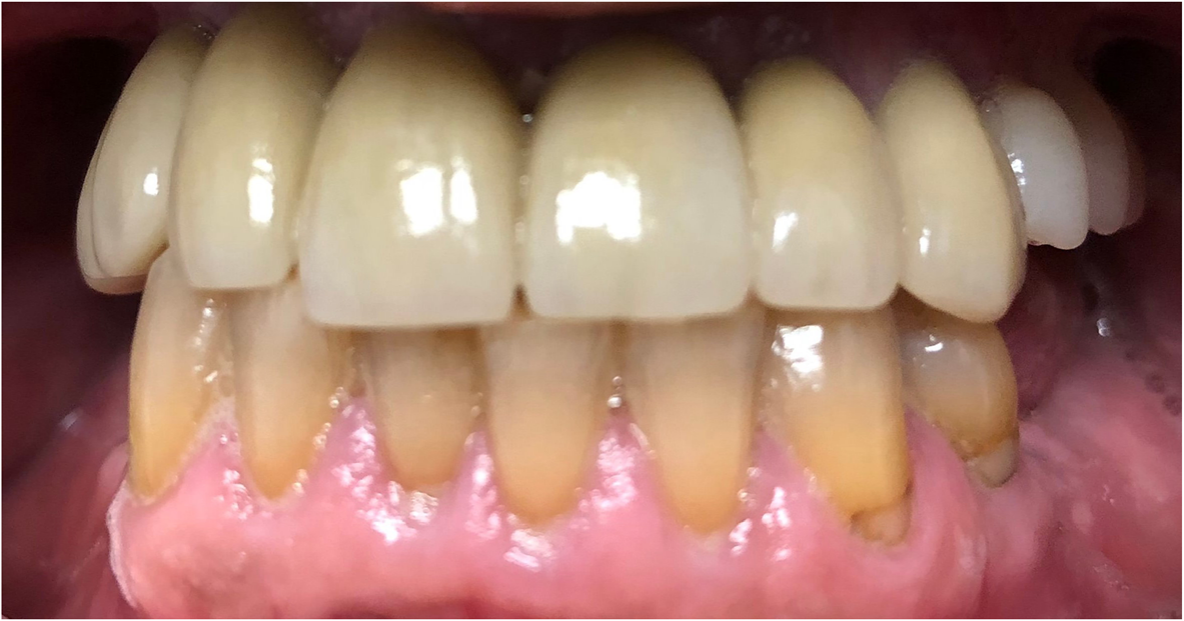
Post-operative frontal view with teeth in occlusion

**Fig. 5:**
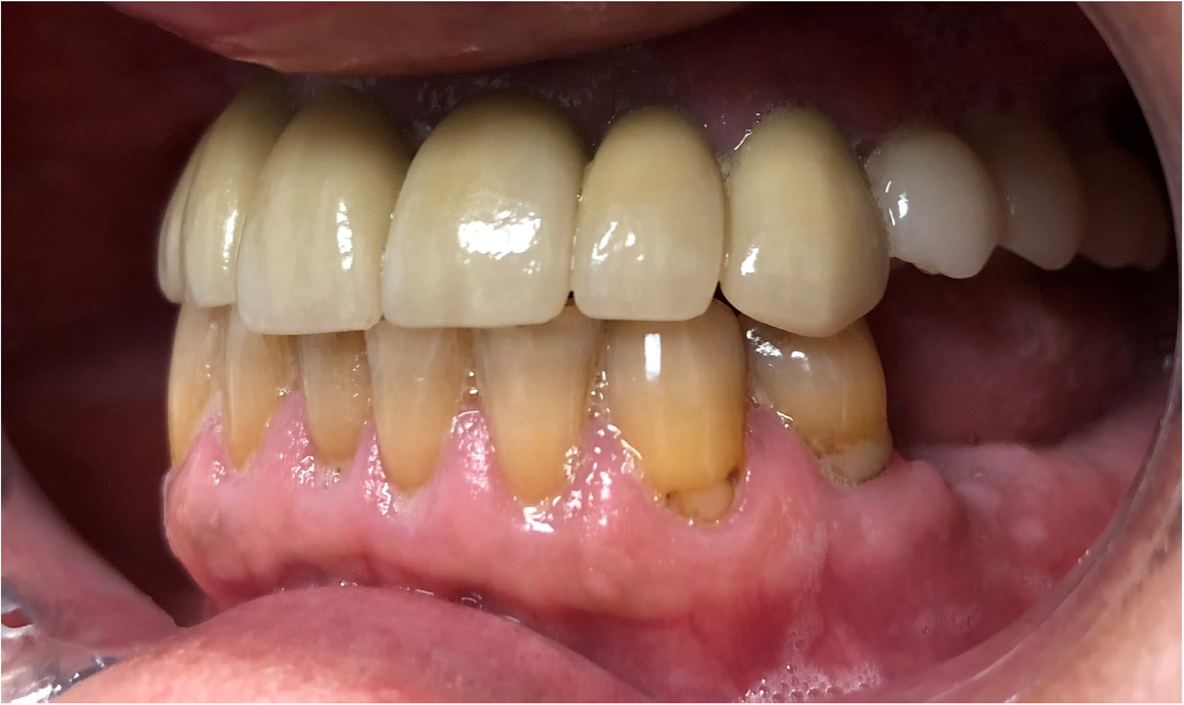
Post-operative lateral view of the left maxillary arch

**Fig. 6:**
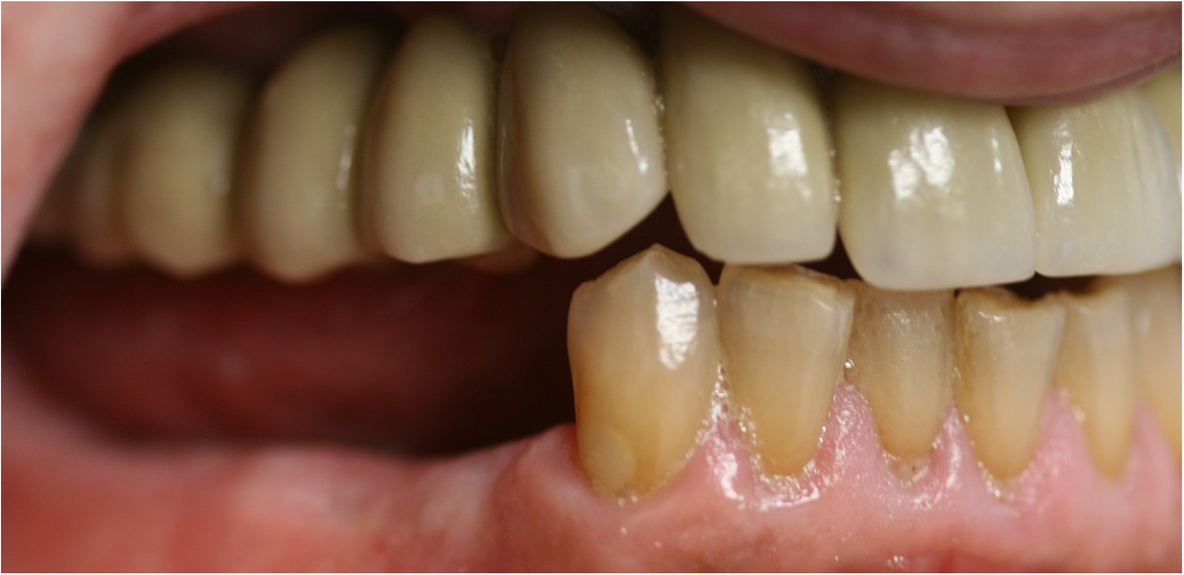
Post-operative lateral view of the right maxillary arch

**Fig. 7:**
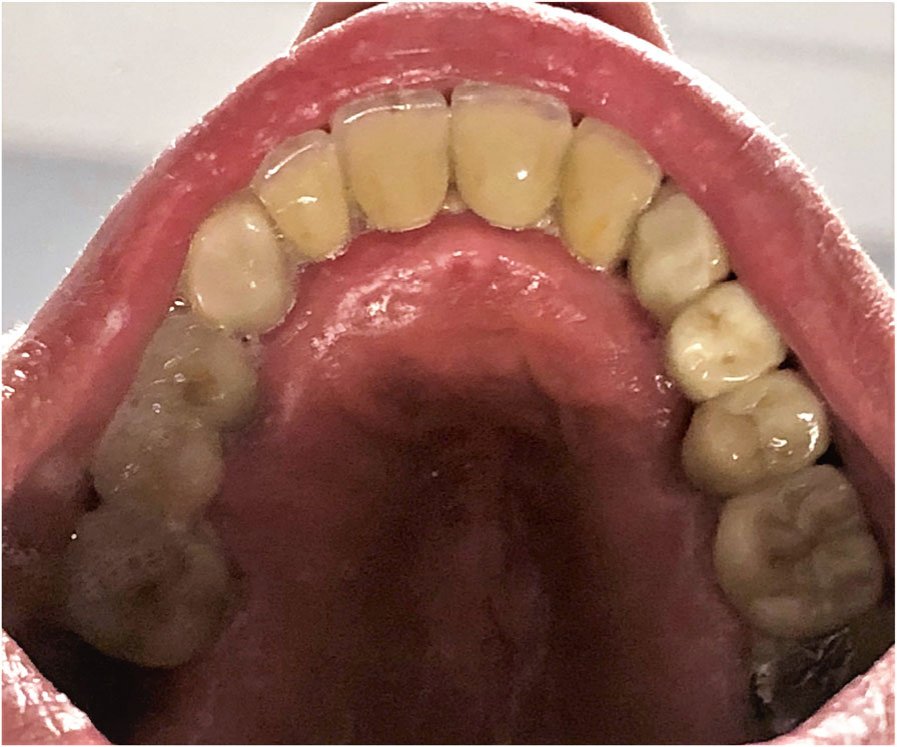
Post-operative occlusal photograph of the maxilla

The standard immediate loading procedures were followed as the patient met the guidelines of a minimum torque value of 35 Ncm. All fixtures placed had intraoperative open tray impressions taken. Impressions were sent to the laboratory, and fabrication of a screw-retained temporary was completed. Temporaries were placed within 24 h of surgery and were torqued at 15 Ncm. Following a 6-month period of functioning in temporary prostheses, final impressions were taken via open-tray technique. He was placed in his final prostheses with no complications. Our patient settled on final prostheses consisting of a four-unit bridge cemented at sites number 3 through number 6; individual crowns placed at sites number 7, number 8, number 9, number 10, and number 11; and a screw-retained, three-unit bridge placed at sites number 12 through number 14 (Figs. 3, 4, 5, 6, and 7). The restorative dentist placed a polymethyl methacrylate (PMMA) prosthesis on the left side, and our patient will transition to his final crowns once he is financially ready.

### Follow-up

Regular hygiene visits show that our OI patient has greatly improved his overall home care routine. No areas of gingival inflammation were found. Probing depths have remained 2–4 mm with no bleeding or purulent drainage at the fixtures sites. There have been no issues with implant mobility, and all healing post-operatively was uneventful.

## Discussion

The vast majority of published articles regarding OI type I revolve around fractures of the long bones and treatment strategies. An extensive literature search for manuscripts detailing the implant therapy for patients diagnosed with OI produced a marginal amount of literature (Table 3). Our case posits that oral restoration is attainable without implant failure for OI type I patients. In OI type I, the collagen produced is of normal quality but in reduced quantities [14]. As a result, OI type I is considered the mildest form of OI with the majority of fractures occurring in childhood and adolescence as the bones continue to grow. Since the collagen is of normal quality, successful osseointegration of implants can be attained with proper planning. To account for poor bone strength, Marx et al. proposed using implants as a “tent-pole” for bone graft to be placed around to consolidate and maintain the graft’s volume [15].

**Table 3:** Reported implant therapy for patients diagnosed with osteogenesis imperfecta

Reference	Age (years)	Gender	OI type	Bone graft augmentation	Number of implants	Implant location	Implant type
Friberg [23]	51	F	N/A**	No	6	Full maxilla	Regular platform TiUnite Brånemark System
Wannfors [18]	30	F	III	Yes	4	Full mandible	OsseoSpeed
Payne [19]	34	F	IV	Yes	11	Full maxilla and mandible	Brånemark Mk III Ti-Unite
Prabhu [24]	32	M	IV	No	11	Full maxilla and mandible	Brånemark titanium bone-tapped
Binger [20]	32	F	N/A**	Yes	5	Full maxilla	ITI dental standard
Lee [21]	43	F	III	Yes	2	Right posterior mandible	Paragon Screwvent internal hexed
Zola [22]	32	M	N/A**	Yes	13	Left and right posterior maxilla and left and right posterior mandible	Not specified

**OI type was not specified

The same factors that must be considered when placing implants in any patient are also pertinent to OI patients. However, extra emphasis should be placed on bone quantity and bone quality. In placing implants for our patient, we ensured that all fixtures attained a final torque value greater than 35 Ncm. Traditional endosseous implants require a bone healing period post-extraction of 3 months for the mandible and 6 months for the maxilla before the implant can be loaded [16]. Innovation in implant technology allows for immediate implant loading following extraction due to design changes that provide a stronger mechanical connection to the surrounding tissue [17]. While these innovations have made implant delivery much more time-effective, primary stability can be challenging in patients with diminished bone quantity and quality. Bone graft augmentation can be utilized to ensure the osseointegration of the implant and has been utilized to achieve positive results in some OI cases [18–22]. However, some cases found successful osseointegration without the usage of bone grafts, including some of the implants placed in our patient [23, 24]. While we were able to successfully deliver implants using synthetic grafting material or no grafting material, other literature utilized autogenous bone from either the ascending ramus [21] or iliac crest [18–20, 22]. In determining the success rate of dental implants, there is a great deal of variability due in part to the varying degrees of bone quality and quantity in the OI subtypes, patient compliance to treatment plans and dental care, and a multitude of other factors typically involved in implant therapy. One retrospective and prospective study cites strong success rates in implant delivery for OI patients with a survival rate between 93 and 100% [25]. Our patient is now 4 years post-placement of his first implant procedure and has been functioning without any issues. The diagnosis of OI type I should not be a contraindication of implant therapy as our case, and others [18–25], have shown. This case differs from other cases in utilizing synthetic grafts to aid in stability and providing another case to illustrate the advancements in implant delivery for patients with bone abnormalities.

## Conclusion

In conclusion, this case shows that implant therapy for patients with OI type I is a viable treatment option with appropriate planning, surgical skill, and routine care. Advancements in the fields of implants, prosthetics, and bone grafting will continue to make implants an increasingly practical treatment option for patients with OI. However, dental practitioners should always take great precaution in ensuring that bone quality and quantity is appropriate to ensure primary stability and successful osseointegration.

## Abbreviation

OI: Osteogenesis imperfecta
